# Current understanding of the molecular mechanisms of circulating permeability factor in focal segmental glomerulosclerosis

**DOI:** 10.3389/fimmu.2023.1247606

**Published:** 2023-09-19

**Authors:** Giuseppe Salfi, Federica Casiraghi, Giuseppe Remuzzi

**Affiliations:** Istituto di Ricerche Farmacologiche Mario Negri Istituto di Ricovero e Cura a Carattere Scientifico (IRCCS), Bergamo, Italy

**Keywords:** FSGS, immunity, permeability factor, circulating factor, post-transplant recurrence, idiopathic nephrotic syndrome

## Abstract

The pathogenetic mechanisms underlying the onset and the post-transplant recurrence of primary focal segmental glomerulosclerosis (FSGS) are complex and remain yet to be fully elucidated. However, a growing body of evidence emphasizes the pivotal role of the immune system in both initiating and perpetuating the disease. Extensive investigations, encompassing both experimental models and patient studies, have implicated T cells, B cells, and complement as crucial actors in the pathogenesis of primary FSGS, with various molecules being proposed as potential “circulating factors” contributing to the disease and its recurrence post kidney-transplantation. In this review, we critically assessed the existing literature to identify essential pathways for a comprehensive characterization of the pathogenesis of FSGS. Recent discoveries have shed further light on the intricate interplay between these mechanisms. We present an overview of the current understanding of the engagement of distinct molecules and immune cells in FSGS pathogenesis while highlighting critical knowledge gaps that require attention. A thorough characterization of these intricate immune mechanisms holds the potential to identify noninvasive biomarkers that can accurately identify patients at high risk of post-transplant recurrence. Such knowledge can pave the way for the development of targeted and personalized therapeutic approaches in the management of FSGS.

## 1 Introduction

Focal segmental glomerulosclerosis (FSGS) is a histological pattern of kidney injury characterized by the obliteration of glomerular capillaries affecting only a portion of the glomerular tuft (segmental) by the deposition of extracellular matrix in some glomeruli (focal) (1). One of the cardinal features of FSGS is the progression of glomerular scarring, with the initial focal and segmental matrix deposition evolving into a widespread form of glomerulosclerosis in the advanced stages (2).

FSGS represents about 20% of cases of nephrotic syndrome (NS) in children and 40% in adults, with an annual incidence between 0.2 and 2.5 per 100,000 individuals. However, sex, geographical, and racial differences should be considered, since FSGS has a higher incidence in male adults and Black individuals (3, 4).

When secondary etiologies of NS cannot be identified, the clinical presentation is designated as idiopathic nephrotic syndrome (INS) (5). The common initiation event in INS is podocyte damage, which ultimately results in podocyte depletion, proteinuria, and progressive kidney disease. Hence all forms of INS are considered part of a larger group of diseases called podocytopathies (6, 7).

Renal biopsy is the foundation of the current classification of INS, as the histological appearance is closely associated with the prognosis and treatment response of the patient (8, 9). In most cases, pathological findings can be classified as either FSGS or minimal change disease (MCD) (10).

Researchers have argued that MCD and FSGS may represent opposite ends of a spectrum, with FSGS as the more severe phenotype associated with a poor prognosis and frequent progression to renal failure (11–13).

## 2 Clinical course and treatment of focal segmental glomerulosclerosis

The age of onset is crucial in determining the clinical course of the disease. A biopsy is usually performed in all adults before initiating treatment (14). However, children with INS are promptly treated with oral prednisolone as the first-line therapy which has a response rate of over 85%, resulting in complete remission of proteinuria and normalization of serum albumin levels (15). A biopsy is only recommended in children with a higher age of onset (>12 years), atypical clinical or biochemical features indicating a secondary form of NS, or in all children who fail to respond to steroid treatment (such cases are defined as steroid-resistant NS or SRNS) (16).

Primary FSGS is characterized by a presentation of full-blown NS of sudden onset and diffuse foot process effacement observed through electron microscopy (17). In contrast, sub-nephrotic or nephrotic-range proteinuria with normal serum albumin requires a evaluation to rule out secondary causes of FSGS (18).

Primary FSGS patients may undergo spontaneous remission, which is very rare and occurs only in less than 5% of the cases (19, 20). Treatment significantly improves patients’ outcomes, as it is associated with an increased likelihood of achieving remission (21, 22).

The first-line treatment for primary FSGS is high-dose oral glucocorticoids (prednisone or prednisolone) (23–25). Some patients might not tolerate prolonged high-dose glucocorticoids, especially considering the extended natural history of primary FSGS. In these cases, the side effects of glucocorticoids could be intolerable (26). Calcineurin inhibitors (CNIs) such as cyclosporine and tacrolimus are effective in reducing or even obviating the need for glucocorticoid therapy (27–29). As such, CNI use is recommended for adults with relative contraindications or intolerance to glucocorticoids (23, 30). The combination of mycophenolate mofetil (MMF) and low-dose prednisolone has been studied as an alternative primary therapy for patients with FSGS and NS. This therapeutic approach has demonstrated comparable efficacy to the conventional high-dose steroid treatment (31). Although the evidence is currently limited, it may be considered as an early treatment option in patients who are more susceptible to the adverse effects of steroids and CNIs, such as those with lower eGFR.

Among all forms of INS, FSGS has the lowest response rate to glucocorticoid therapy (32), with steroid resistance being observed in 26-80% of patients across various studies (33–36). Notably, adults tend to respond much less favorably to corticosteroids than children (37).

Additionally, among the initial steroid-sensitive (SS) patients, less than 50% can maintain stable remission (19, 21) Relapses are frequent and if they occur during therapy or within 2 weeks of discontinuing prednisone or prednisolone, the disease is considered steroid-dependent (23).

In cases where patients with FSGS exhibit resistance to glucocorticoid therapy, genetic testing should be considered to rule out genetic forms of the disease (23).

Numerous secondary therapeutic options are available for steroid-resistant primary FSGS. Currently, the most robust evidence supports the use of CNIs for at least six months, rather than continuing with glucocorticoid monotherapy or stopping treatment altogether (23, 33, 38–40).

MMF has also demonstrated effectiveness in treating steroid-resistant FSGS, although the clinical outcomes have shown less significance compared to CNIs (41, 42).

For patients with either steroid- and CNI-dependent or resistant FSGS, the usage of chimeric or human anti-CD20 antibodies (i.e. rituximab and ofatumumab) is well established and guarantees prolonged maintenance of remission status (43, 44).

In an advanced immunosuppressive therapy-resistant setting, several novel treatments have been trialed with promising results. These include monoclonal antibodies such as various anti-TNFα antibodies (such as adalimumab) (45), as well as extracorporeal plasma therapy (46). Notably, apheresis treatment has also exhibited promising efficacy in INS patients unresponsive to immunosuppressive regimens (47).

In addition, sparsentan, which is a dual endothelin and angiotensin receptor blocker, has also been proposed to reduce proteinuria in patients with FSGS and nephrotic syndrome (48). However, it is important to note that sparsentan only serves as a supportive treatment aimed at reducing proteinuria in FSGS patients, rather than specifically targeting the underlying pathogenetic mechanism of the disease.

While it can be argued that these therapies can be effective in slowing the progression of the disease, there are notable limitations and areas for improvement. First, the use of multiple drugs can have a significant impact on patient quality of life due to their side effects (49, 50). Another important consideration is that FSGS consistently worsens over time, regardless of the treatment used, and can result in end-stage kidney disease (ESKD) in approximately half of the patients with nephrotic-range proteinuria within 3-8 years (51).

### 2.1 End-stage kidney disease and transplantation in focal segmental glomerulosclerosis

A recent analysis conducted in the United States reveals that FSGS is the leading cause of primary glomerular disorder resulting in ESKD, accounting for 3.7% of all cases of ESKD (52). Patients with ESKD due to primary FSGS are often candidates for renal transplantation (53).

On the other hand, while transplantation should not be withheld based solely on a primary FSGS diagnosis, clinicians must ensure that patients are fully informed of the elevated risk of disease recurrence after transplantation. Indeed, post-transplant FSGS recurrence is reported in approximately 30% of transplants, ranging from 9% to 55% among different studies (54, 55).

The average time for recurrence of FSGS in the transplated kidney is 2-6 days, but it can sometimes develop even within minutes to hours after transplantation (56).

Some patients are offered a second kidney transplant, however, the risk of recurrence in patients with FSGS who have previously lost a transplant due to recurrent disease is significant, estimated to be around 80% (57).

Plasmapheresis and rituximab are most commonly frequently used to treat recurrent FSGS, but their effectiveness is limited to a small percentage of patients (55).

Prophylactic pre-transplant rituximab might even play a role in preventing FSGS recurrence (58–60), while results with plasmapheresis are contrasting in the pre-transplantation setting. However, due to inconsistent results and the lack of randomized controlled trials, the use of pre-transplant therapy to reduce the risk of disease recurrence is not recommended and should be avoided (54, 61).

To summarize, treating patients with recurrent FSGS proves to be complicated, as not a single approach has been consistently effective. Despite experimental studies providing valuable insights into the pathophysiology of the disease, treatment options for affected patients remain largely empirical.

**Figure 1:**
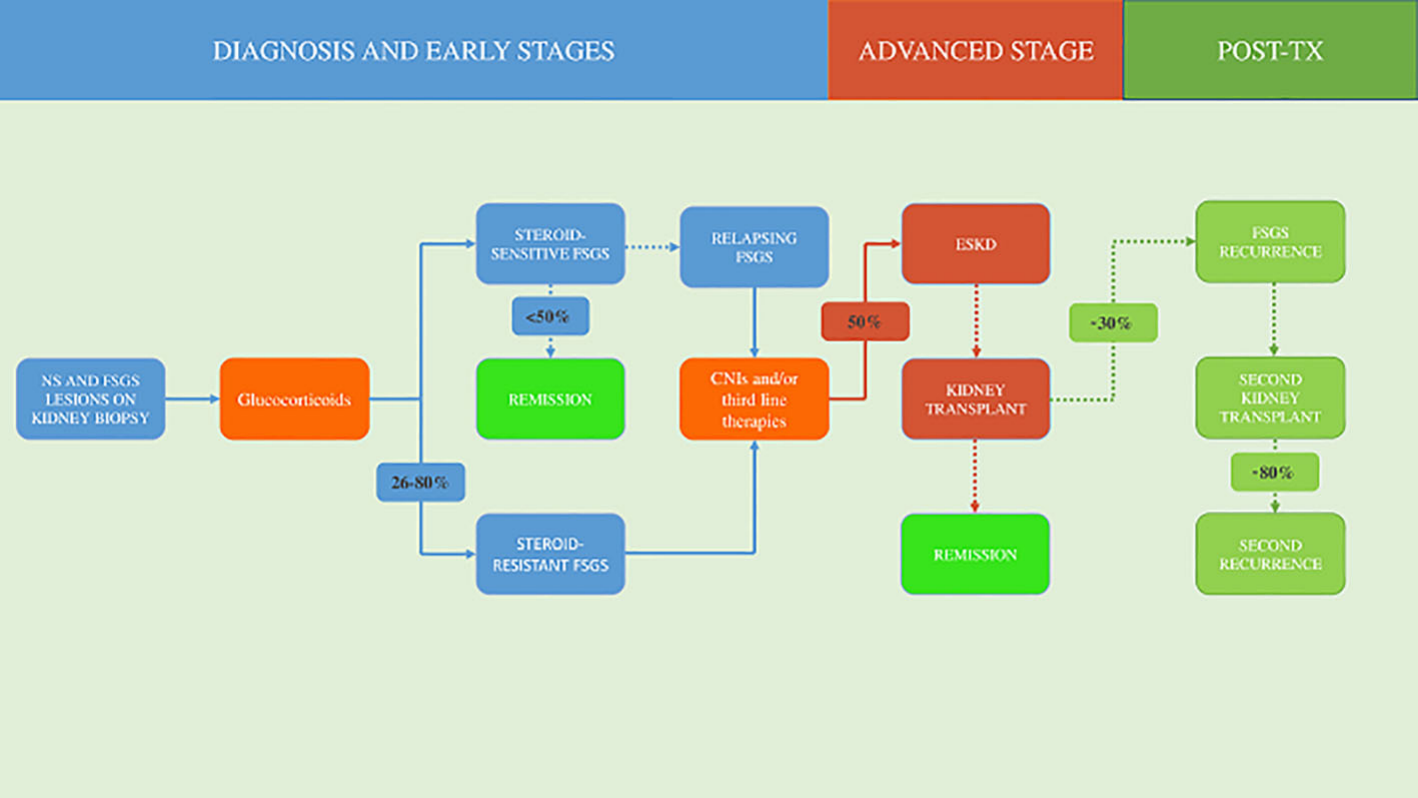
Natural history and treatment of focal segmental glomerulosclerosis. NS, nephrotic syndrome; FSGS, focal segmental glomerulosclerosis; CNIs, calcineurin inhibitors; ESKD, end-stage kidney disease; TX, transplantation.

FSGS remains an intricate disease to provide treatment and continues to have a devastating and unrelenting impact on the lives of those affected (**Figure 1**).

## 3 Classification of focal segmental glomerulosclerosis

FSGS classification can be approached through two main frameworks: etiologic and morphologic, both of which hold clinical and prognostic significance (62).

### 3.1 Pathologic classification

The established morphological classification, known as the Columbia Classification, distinguishes five different types of FSGS lesions based on their histologic presentation: collapsing, tip, cellular, perihilar, and not otherwise specified (NOS) (63). The FSGS NOS variant – defined by the exclusion of all other categories - is the most common among both the pediatric and adult populations (64, 65). Interestingly, as previously discussed, lesions tend to change over time, with other variants evolving into a NOS phenotype, especially as the kidney approaches ESKD (12, 66).

The categorization of the FSGS form according to the Columbia classification associates with the clinical course of the disease (67, 68). Reports indicate that patients with the collapsing variant of FSGS have worse outcomes and less frequent remission of proteinuria, while those with the tip variant respond better and more frequently to various immunosuppressive treatments (68–70).

However, the Columbia histologic variant does not predict the risk of FSGS recurrence in renal allografts, and interestingly, different variants can be seen before and after transplantation in the same patient (71).

### 3.2 Etiologic classification of FSGS

Traditionally, FSGS forms have been classified into primary, genetic, or secondary categories (2, 18).

The term “primary” has often been used interchangeably with “idiopathic” to describe a condition without a known genetic or secondary cause (23). However, genetic testing is not routinely performed in most FSGS cases due to its limited cost-effectiveness (72, 73). Therefore, it can be argued that FSGS caused by known genetic mutations could also be classified as primary FSGS, considering that the effect of the mutation primarily involves podocyte injury (74).

In addition, a fourth category of “FSGS of undetermined cause” has been proposed for those cases where no clear etiology can be identified, and for patients exhibiting proteinuria without NS and no diffuse foot process effacement on electron microscopy (but of course, FSGS lesions are seen on light microscopy) (23, 75–77).

As a result, some authors recommended avoiding the term “idiopathic” to describe any form of FSGS, to prevent confusion (23, 78). Instead, the definition of “primary FSGS” should be limited to those cases of FSGS where the pathogenesis is presumably associated with an unknown “circulating” or “permeability factor” (79, 80).

Hence, recent efforts to reclassify FSGS based on its pathogenesis have identified four different categories: secondary forms (maladaptive FSGS, drug-induced FSGS, viral-induced FSGS, and FSGS lesions superimposed on other glomerular diseases), genetic disorders, permeability factor-related FSGS (i.e., primary FSGS), and FSGS of undetermined cause (74, 78, 81).

#### 3.2.1 Genetic FSGS

Although the aim of this review is to examine the nature and the complex mechanisms underlying the pathogenesis of primary FSGS and its recurrence after transplantation, valuable insights can be gained from studying the abnormalities associated with genetic forms of FSGS.

The term “genetic FSGS” encompasses all monogenic etiologies of FSGS, including primary forms with nephrotic proteinuria, and secondary forms characterized by renal anomalies such as congenital developmental anomalies, nephronophthisis, chronic tubulointerstitial disease, or proximal tubulopathies (82). Furthermore, monogenic FSGS may present as a kidney-specific condition or as a syndromic disorder with extrarenal manifestations, as exemplified by Alport syndrome, Pierson syndrome, and nail-patella syndrome (83–86).

While genetic mutations have been identified as the cause of FSGS in a minority of patients (involving around 20–30% of individuals with SRNS and FSGS) (87), their prevalence appears to be higher in those with congenital or infantile-onset disease. A study found that 100% and 57% of patients with these forms of FSGS respectively had a genetic cause identified (88).

The prevalence of monogenic forms of FSGS seems to decrease with an older age of onset (89, 90). In children, mutations in genes such as NPHS1 and NPHS2, which encode for structural proteins nephrin and podocin respectively, as well as the WT1 gene, which is involved in podocyte development, have been identified as the major genetic causes of FSGS, as detection rates of up to 24% have been reported (1, 9, 91). The prevalence of monogenic FSGS in adult patients is not well established, as genetic testing is mainly conducted in individuals with early-onset disease, immunosuppression-resistant cases, positive family history, or syndromic manifestations (23, 82). Recent research has shown rates of genetic diagnoses ranging from 11% to 21.3% among various cohorts of adult FSGS and/or SRNS patients (92–95).

A wide array of mutations in more than 50 different genes has been identified in pediatric- and adult-onset familial FSGS. Among these genetic variations, more than 20 mutations have been detected in proteins related to slight diaphragm proteins, cytoskeletal structural and regulatory proteins, nuclear pore complex proteins, cell membrane-associated proteins, and glomerular basement membrane proteins genes, and are involved in the development of renal-limited forms of FSGS. Conversely, 34 mutations have been linked to FSGS within a systemic/syndromic context (18, 73, 96).

Among individuals with recent African ancestry, the presence of genetic risk variants in the gene encoding apolipoprotein L1 (APOL1) has been identified as a significant factor associated with an increased susceptibility to developing FSGS (97). Interestingly, an APOL1 inhibitor, inaxaplin, has demonstrated efficacy in reducing proteinuria in FSGS patients carrying two APOL1 variants (98).

Careful clinical assessment and genetic testing are crucial components of FSGS management, as genetic FSGS has a distinct clinical course, which typically results in primary resistance to therapy (18, 99). Moreover, in adults with FSGS who are undergoing kidney transplantation, genetic testing may offer valuable prognostic information regarding transplant outcomes (23). Genetic forms of the disease have shown a significantly lower rate of FSGS recurrence after transplantation, with some studies reporting no cases of post-transplant FSGS recurrence (100, 101).

Apart from one case report describing post-transplant FSGS recurrence in a young girl carrying a WT1 mutation (102), only mutations of NPHS2 have been associated with FSGS recurrence (101, 103).

Despite extensive research on the topic, there is currently a dearth of data on the impact of genetic mutations associated with adult-onset FSGS, including but not limited to *INF2, ACTN4, TRPC6*, and *PAX2*, on the risk of disease recurrence post-transplantation. As such, further investigation is warranted to elucidate the potential prognostic value of genetic testing in adult FSGS patients undergoing kidney transplantation. In addition, assessing the presence of a genetic mutation for FSGS in living-related donors can be crucial to predicting the risk of post-transplant disease recurrence, particularly in individuals who may not exhibit any symptoms during evaluation (23).

Therefore, recognizing patients with genetic FSGS can significantly enhance personalized clinical management and lead to improved long-term outcomes.

Furthermore, dissecting the mechanisms underlying the development of genetic FSGS may play a crucial role in directing research toward a better understanding of the pathogenesis of non-genetic primary FSGS forms, including those related to permeability factor(s).

## 4 Permeability/circulating factor-related FSGS

The pathogenesis of those forms of FSGS characterized by a lack of identifiable secondary causes or genetic mutations has been attributed to one (or more) molecule(s) with an extra-renal origin, produced in a systemic context, and located in patients’ serum. These molecules are known as “circulating factors” or “permeability factors” and are believed to selectively target and damage the glomerular barrier, particularly the visceral epithelial cells (i.e. the podocytes), leading to increased permeability and massive proteinuria, which are hallmarks of FSGS. As previously discussed, this pathogenic mechanism is also applicable to all forms of INS, including those with characteristics of MCD on kidney biopsy (6, 79, 104). As a result, some authors refer to these conditions as a single “circulating factor disease” (105–107).

The identity of the circulating factor in the blood of idiopathic INS patients has been a topic of interest in the nephrology community for several decades now. Despite extensive research efforts, the precise identity of this factor remains elusive, leaving much to be revealed about its biological properties and pathological significance.

An important concept that must be highlighted is that currently, only the forms of FSGS that recur rapidly after a kidney transplant can be attributed to a circulating permeability factor (23). This involves an extremely rare population of patients, which explains the fact that the vast majority of the studies searching for a pathogenetic circulating factor actually included mostly or only patients with steroid-resistant FSGS and no history of kidney transplantation. However, it should be noted that this population has a 70% probability of disease remission after transplantation is performed, meaning that they most likely don’t have a circulating factor disease (54–56). This is the most significant limitation of such studies and can affect the generalizability of the research findings.

## 5 Historical background and proofs of the presence of a circulating factor in INS

The history of the circulating permeability factor’s theory dates back to 1954 when Gentili et al. conducted an ethically questionable study in which plasma from infants with INS was administered to healthy individuals, who subsequently developed transient proteinuria. However, due to the lack of techniques available to study plasma composition at the time, the authors’ understanding of the phenomenon was limited. The authors attributed the results to the presence of abnormally small proteins in the plasma of the patients, as it was believed to be the most reasonable pathogenetic model for INS at the time. However, they also went on to propose, for the first time in literature, a novel theory: the presence of a serum circulating factor, which they termed a “toxic factor” or “nephrotoxin”, able to damage the glomerular filter barrier, causing the disease (108).

Nowadays, researchers agree that the strongest evidence for the existence of extra-renal circulating permeability factors and their role in chronic primary glomerulopathies comes from the clinical observations of the recurrence of FSGS after kidney transplantation (109, 110). This phenomenon was first reported in 1972, in a case involving two children and a young adult with INS and MCD on initial kidney biopsy. The conditions of the patients worsened over time despite the administration of high-dose corticosteroids in combination with other immunosuppressive agents. ESKD manifested within 2-6 years. Subsequent transplantation resulted in the patients exhibiting disease recurrence within 1-5 months, characterized by the development of nephrotic-range proteinuria. Histological examination of their kidney graft biopsies revealed a pattern consistent with the diagnosis of FSGS, without any indications of graft rejection (111).

In 1974, Robert J. Shalhoub introduced a seminal theory known as “Shalhoub’s hypothesis”, which postulated an immune system abnormality as a potential underlying cause of FSGS. According to Shalhoub, this abnormality primarily affected the functionality of T cells. He suggested the existence of a “circulating chemical mediator” as the culprit for damaging the glomerular basement membrane. Shalhoub’s hypothesis was supported by several observations, including the absence of antibody deposits in FSGS patients’ glomeruli, the effectiveness of steroids and immunosuppressive drugs in inducing remission, and the co-occurrence of FSGS and NS in some cases of Hodgkin’s lymphoma (112). The presence of a proteinuria-inducing factor in the serum of FSGS patients was confirmed by Zimmerman SW et al. in 1984. Serum collected from a patient who experienced NS and FSGS recurrence following two cadaveric renal transplants was infused into rats inducing a significant increase in protein and rat albumin excretion (113). A decade later, reports about plasmapheresis performed in recurrent FSGS patients were first published. They showed a transient decrease or abolishment in patients’ proteinuria, resulting in partial or complete remission, hence providing further evidence of a pathogenetic factor circulating in that group of patients (114, 115).

In 1996, Savin VJ et al. introduced an *in vitro* model to measure the effect of the FSGS permeability factor on the permeability of the glomerular barrier. The model relied on the usage of isolated rat glomeruli that were incubated in isotonic bovine serum albumin. Exposure of the isolated rat glomeruli to the serum of patients with recurrent FSGS resulted in reduced glomerular swelling, due to increased glomerular permeability and dissipation of the oncotic gradient. The exposure of the glomeruli to the sera of patients who experienced FSGS post-transplant recurrence increased glomerular permeability to albumin (Palb) significantly more than the sera of patients with non-recurrent FSGS. A Palb cut-off of 0.50 predicted recurrence with a sensitivity of 60% and specificity of 95% (116). This model supported the causative role of a circulating factor in FSGS patients and has been used since then to make well-established hypotheses about its nature. However, its low replicability and lack of application in recent FSGS research should be noted. Most of the findings that resulted from its application were obtained by the same group that developed it, and little information can be found about its usage in the literature. Finally, all findings from the model should be subjected to *in vivo* evaluation due to their *in vitro* nature.

The most impactful evidence of the fact that the pathogenetic factor of primary FSGS is circulating and extra-renal was provided in 2012 by the groundbreaking work of Gallon L et al. (117). In their study, a 27-year-old man with ESKD caused by primary FSGS received a kidney transplant from his healthy sister. Despite undergoing seven plasmapheresis sessions and receiving standard immunosuppressive therapy, the patient exhibited a significant increase in proteinuria on the second day after transplantation. Histological analysis of the kidney biopsy confirmed the recurrence of FSGS. Subsequently, due to the persistent disease and associated complications, the renal allograft was removed. In a pioneering and brave decision, the authors sought consultation with the hospital ethics committee and obtained informed consent from the initial recipient to donate the kidney transplant to another willing patient on the transplant waiting list. This kidney transplant was successfully performed on a 66-year-old patient with ESKD resulting from type 2 diabetes mellitus, utilizing the failing allograft from the previous patient who had experienced early post-transplant FSGS recurrence. Remarkably, following the retransplantation, the allograft rapidly regained full functionality, with serum creatinine and proteinuria levels returning to within the normal range values. This groundbreaking trial unequivocally confirmed the presence of a distinct extrarenal circulating permeability factor exclusive to patients with recurrent FSGS.

The trial was later replicated by Kienzl-Wagner et al., as described in a study published in 2018, strengthening its scientific significance. In this most recent trial, after a 5-year-old boy experienced an immediate and fulminant recurrence of FSGS on a deceased donor renal graft, the allograft was retrieved from him 27 days after the transplant and implanted into a 52-year-old second recipient with vascular nephropathy. After retransplantation, the allograft regained perfect functioning, which persisted for 3 years after transplantation (118).

In summary, the concept of a circulating permeability factor affecting podocyte shape and function is believed to be the cause of primary FSGS. This concept is supported by several observations, the most relevant ones include: 1) after kidney transplantation, around 30% of patients with FSGS develop massive proteinuria within hours to days after transplantation, followed later on by typical FSGS histological lesions (54–56); 2) pre-emptive plasmapheresis reduces the risk of FSGS recurrence after transplantation (119); 3) the perfusion of rat glomeruli with the plasma of patients with FSGS induces an increased glomerular permeability to albumin (116); 4) a kidney graft removed from a primary FSGS patient can be successfully transplanted in a patient with different kidney disease with normalization of proteinuria and glomerular filtration rate (GFR) (117, 118).

Studies on the pathogenesis of INS have indicated several different molecules as possible permeability factors. However, the effect of such molecules on the pathogenesis of INS and primary FSGS remains elusive.

Subsequently, this review delves into a comprehensive analysis of the evidence that underpins the main circulating factors and immunological mechanisms proposed to contribute to the pathogenesis of primary and post-transplant recurrent FSGS. Through a thorough examination of these factors, this review aims to shed light on their potential role in the development and progression of these conditions.

## 6 T cells and related cytokines

Since the publication of Shalhoub’s seminal paper in 1974 until recently, INS has been predominantly attributed to a T-cell disorder. Shalhoub’s hypothesis was initially supported by numerous distinct observations: the remission of INS following measles infection, the absence of evidence for a humoral antibody response in INS, the rapid resolution of proteinuria with immunosuppressive drug therapy, the occurrence of MCD in Hodgkin’s disease patients, and the increased susceptibility of untreated patients to pneumococcal infections (112).

However, it is essential to acknowledge that those initial lines of evidence supporting the notion of T-cell dysfunction (and thymus malfunction, as suggested by Shalhoub) as fundamental factors in the pathogenesis of FSGS are now considered outdated. The interpretation of the same findings has evolved, as they could also support the hypothesis of alternative mechanisms in the pathogenesis of FSGS, including the possibility of a B-cell-mediated disease. Therefore, it is imperative to reevaluate the role of T cells in the disease, taking into account recent insights and advancements (120).

Building upon Shalhoub’s hypothesis, extensive research has been completed to investigate this phenomenon. Subsequent studies showed that T cells from patients with MCD could secrete a glomerular permeability factor, but this factor was not conclusively identified (121, 122).

Untreated NS exhibited higher absolute T cells and T-cytotoxic (CD8^+^) lymphocytes compared to healthy controls (123). Further studies revealed that CD8^+^ T cells from steroid-resistant nephrotic syndrome (SRNS) patients are clonally expanded (124).

Cytokines secreted by T cells have been therefore proposed as potential permeability factors in INS.

### 6.1 Interleukin-13

Various studies suggested a putative role for T-helper 2 (Th2) cytokines, mainly interleukin-13 (IL-13), in the pathogenesis of INS. T cells from patients with INS have been observed to exhibit spontaneous production of IL-13, whereas B cells express the IL-13 receptor (125). Notably, Yap et al. demonstrated upregulated IL-13 gene expression in both CD4^+^ and CD8^+^ T cells in children with SSNS during relapses (126). The putative involvement of IL-13 in the pathogenesis of INS was further highlighted by the finding that rats overexpressing IL-13 developed nephrotic proteinuria and podocyte foot process fusion, closely resembling key features of human MCD (127). In addition, significantly increased levels of Th2 cytokines (IL-4, IL-5, IL-10, IL-13) were found in the urine samples of MCD patients with frequent relapses (128). However, it should be noted that increased levels of IL-13 in other pathological conditions, such as asthma, psoriasis, and allergic dermatitis, do not typically result in proteinuria. Finally, some studies conducted on humans did not show the same findings (129).

### 6.2 Tumor necrosis factor-alpha

Some Th1 cytokines have also been implicated in the pathogenesis of INS. Among them, tumor necrosis factor-alpha (TNF-α) holds a special interest.

The upregulated expression of TNF-α in podocytes following treatment with sera from FSGS patients revealed the presence of serum biomarkers, still unknown, that can induce podocyte injury through the activation of the TNF-α pathway (130). Comparative analysis demonstrated elevated TNF-α serum levels in NS patients compared to normal controls, with higher levels observed in patients with SRNS compared to those with SSNS (131). The role of TNF-α in the pathogenesis of SRNS has therefore been investigated in clinical studies with the chimeric or humanized anti-TNFα antibodies (i.e. infliximab, etanercept, and adalimumab). Initially, a few case reports demonstrated remission of severe refractory SRNS in native kidneys and SRNS relapse after kidney transplantation following the administration of anti-TNF-α antibodies (132, 133). Subsequent investigation revealed the activation of the TNF pathway in cultured podocytes exposed to serum from FSGS patients in just 21% of the cases (134). These findings were consistent with the results of a phase 1 clinical trial conducted among FSGS patients, using adalimumab. Although it was not the primary goal, proteinuria was observed to be significantly reduced by treatment in 40% of patients, thus proving that TNF-α may play a crucial role in the disease in a subgroup of FSGS patients (135). Finally, recent observations from the NEPTUNE network investigators suggest that patients with activation of the TNF pathway might be at higher risk for rapid renal function deterioration (136).

### 6.3 T-regulatory and T-helper 17 cells

The involvement of T-helper 17 (Th17) cells and the dysregulation of T-regulatory (Treg) cell function are additional elements implicated in the pathogenesis of INS.

In patients with relapsing idiopathic MCD, Treg exhibited impaired suppressive capacity on conventional T cell proliferation (137). Moreover, higher Treg levels have been observed in patients with SSNS compared to SRNS (138), and lower baseline levels of Treg were associated with an increased risk of early relapse (i.e., within 1 year) in SRNS patients treated with rituximab (139). Specifically, CD45RO^+^ memory Tregs were shown to be lower in steroid-dependent/frequently-relapsing NS patients who experienced relapse after treatment with rituximab (140).

Another study showed that MCD patients with NS have a higher Th17/Treg ratio, which correlated with increased proteinuria and decreased albumin levels. Notably, patients showed a reduction of the Th17/Treg ratio after successful treatment (141). *In vitro* studies further revealed that treatment of podocyte cultures with Th17 cell supernatants from healthy donors and plasma from NS patients led to increased podocyte motility via the c-Jun N-terminal kinases (JNK) pathways (142).

Furthermore, the development of NS and MCD or FSGS within the context of Immune Dysregulation Polyendocrinopathy, Enteropathy, and X-linked (IPEX) syndrome strengthens the notion of a potential involvement of deficient Tregs in the pathogenesis of INS. IPEX syndrome occurs due to mutations in the forkhead box P3 (FoxP3) gene, resulting in impaired Treg maturation (143, 144).

Interestingly, IL2 up-regulates the activity of Tregs and has been trialed as a potential therapeutic agent in INS resistant to multiple treatment lines. Nevertheless, clinical trials did not demonstrate significant clinical benefits from its use (145).

The accumulated evidence proves the critical involvement of T cells in FSGS and INS pathogenesis. While it remains unclear if T cells are directly responsible for the production of the pathogenetic circulating factor(s), the literature clearly highlights their crucial role as potential sources or essential mediators of the disease.

## 7 Cardiotrophin-like cytokine factor-1

A prominent candidate molecule in the pathogenesis of FSGS is Cardiotrophin-Like Cytokine Factor-1 (CLCF-1). CLCF-1 is a member of the IL-6 family of cytokines and shares significant structural similarities with cardiotrophin-1 and ciliary neurotrophic factor. The mature form of CLCF-1 is estimated to have a molecular weight of 22 kDa. The discovery of CLCF-1 dates back to 1999. Several alternative names have been attributed to this protein, including cardiotrophin-like cytokine 1 (CLC-1), B cell stimulatory factor-3 (BSF3), and novel neurotrophin-1 (NNT-1). It is expressed in various tissues, including lymph nodes, spleen, bone marrow, peripheral blood lymphocytes, ovary, placenta, kidney, pituitary, and fetal liver, among others (146–148).

CLCF-1 exhibits a broad range of functional involvement beyond renal processes. Studies have demonstrated its significance in neural differentiation and survival, potentially acting as a ligand for CNTFRα to support neural growth (149). Furthermore, it has been implicated in protecting against osteoporosis (150, 151). Notably, researchers have also suggested its potential as a biomarker in blood and solid cancers (152, 153).

Interestingly, CLCF-1 may be obtained from activated T cells *in vitro* and is able to stimulate B cell proliferation and IgG production (154).

The recognition of CLCF-1 as a plausible permeability factor in primary FSGS is the culmination of over two decades of research conducted by Virginia J. Savin’s team. As previously documented, their investigation began with an initial study where isolated rat glomeruli were subjected to FSGS plasma or serum. The findings revealed an augmented permeability to albumin in rat glomeruli exposed to FSGS samples when compared to those treated with control samples, specifically in response to an oncotic albumin gradient (116). Through systematic investigation of the biochemical characteristics of the active fraction of FSGS plasma, Savin’s research team identified the permeability factor through the implementation of galactose affinity chromatography and mass spectrometry techniques. This investigative process revealed the potential involvement of a small protein with an estimated molecular weight < 30kDa, exhibiting a strong affinity for galactose, which likely contributes to the observed increase in permeability. Finally, CLCF-1 was identified within the active fraction isolated via galactose chromatography in plasma samples obtained from individuals with recurrent FSGS (79, 155).

Using the previously described *in vitro* model, the same group showed that recombinant CLCF-1 exhibited a dose-dependent ability to heighten Palb. Conversely, the introduction of an anti-CLCF-1 monoclonal antibody effectively prevented the Palb increase induced by CLCF-1 (156).

**Figure 2:**
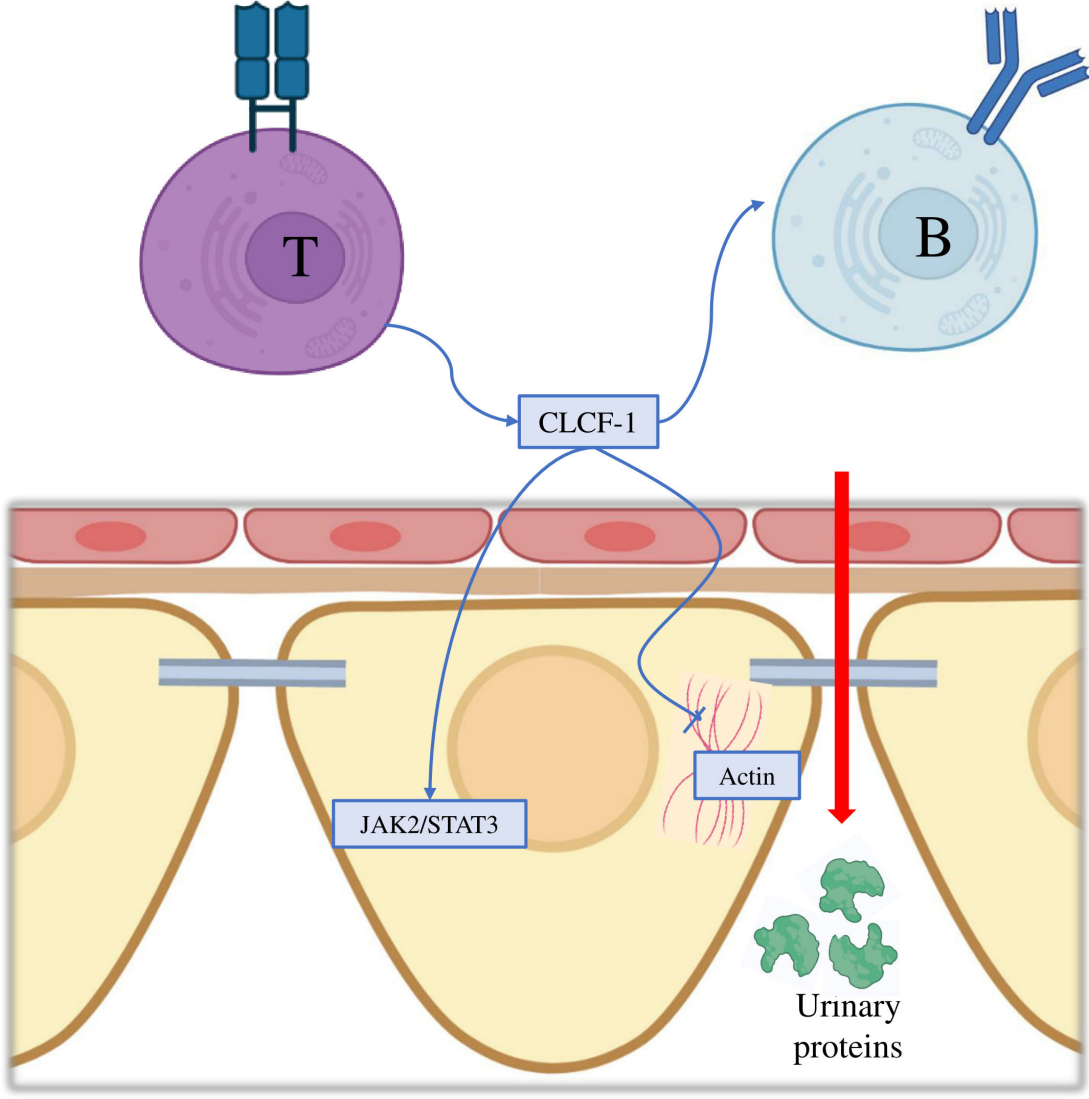
Molecular mechanisms of Cardiotrophin-Like Cytokine Factor-1 in the pathogenesis of focal segmental glomerulosclerosis. CLCF-1, Cardiotrophin-Like Cytokine Factor-1; JAK2, Janus kinase 2; STAT3, Signal transducer and activator of transcription 3.

The authors showed that CLCF-1 acts at the glomerular level via the activation of the Janus kinase/Signal transducer and activator of transcription (JAK/STAT) pathway. Podocytes, indeed, particularly express JAK2 and STAT3, which are activated through tyrosine phosphorylation by CLCF-1. This was evidenced by the fact that preincubation with JAK2 or STAT3 inhibitors impeded the effects of CLCF-1 and FSGS serum on increasing Palb. Notably, incubation of cultured murine podocytes with CLCF-1 led to alterations in the cytoskeleton configuration, characterized by filopodia retraction and attenuation of basal parallel actin filaments (156, 157) (**Figure 2**).

Furthermore, the study revealed a significant increase in urine albumin/creatinine ratio upon CLCF-1 injection in mice, thereby confirming its ability to induce proteinuria independently. However, it is important to note that this finding is subject to certain limitations. The degree of albuminuria/proteinuria observed in the mice was lower than that typically seen in classical human FSGS. The authors hypothesized that this discrepancy may be due to mice exhibiting resistance to developing proteinuria and/or differences in the activated pathways between mice and humans in renal disease. They further suggested that additional plasma components might be required to induce maximal proteinuria and accurately replicate renal disease resembling human FSGS.

A 2010 literature review (79) reports that comparative studies have been conducted between patients with recurrent FSGS and healthy control subjects regarding the levels of CLCF-1. According to the authors, the analysis revealed a remarkable elevation in CLCF-1 concentrations in the former group, reaching approximately 100 times higher levels.

Moreover, a recent study conducted by Chebotareva et al. investigated the levels of CLCF-1 in sera obtained from patients diagnosed with various chronic glomerulopathies, reporting no significant variation in CLCF-1 levels between NS patients and those with other glomerulopathies. Serum CLCF-1 levels correlate with proteinuria and serum lipids in patients with NS. Furthermore, the study observed that CLCF-1 levels remained unaffected by the temporal relationship with immunosuppressive treatments, as similar levels were observed in samples obtained before and after treatment. However, it is important to acknowledge that a significant limitation of this study is the lack of a healthy subject control group. As a result, drawing conclusions regarding the normal values of CLCF-1 in healthy subjects and the relative differences compared to the examined patients is not feasible based on this study alone. To obtain a comprehensive understanding of CLCF-1 role and its levels in both healthy individuals and patients with glomerulopathies, further research incorporating a healthy control group is necessary (158).

Building upon the emerging evidence, multiple research groups have sought to target this presumed circulating factor in a human patient setting.

In a study conducted by Savin et al. in 2008, it was demonstrated that galactose exhibited a high affinity for the permeability factor (later identified as CLCF-1) and effectively inactivated its activity *in vitro*. The proposed rationale for galactose therapy in FSGS patients involves the presence of galactose-binding sites on the permeability factor which interact with galactose of the glomerular glycocalyx, ultimately leading to proteinuria. Supplementation with galactose may therefore block the permeability factor’s binding sites, turning the factor inactive, and promoting clearance of the permeability factor–galactose complex through the liver metabolism (155).

However, when tested on a patient with post-transplant FSGS recurrence, galactose supplementation did not result in an improvement in proteinuria. The authors justified this by suspecting the presence of already established irreversible glomerular injury. Two subsequent case studies reported complete or partial remission of NS with galactose therapy (159, 160).

In an attempt to replicate those findings, Sgambat K et al. conducted a prospective clinical trial to investigate the effect of oral galactose on circulating factor activity and proteinuria in seven children with idiopathic SRNS (including two patients with post-transplant FSGS recurrence). Unfortunately, while galactose administration decreased the permeability factor activity, it failed to improve proteinuria in any of the patients (161).

In another study conducted in 2015, it was found that among seven patients with immunosuppressive-resistant FSGS who received galactose treatment, only two individuals achieved the primary endpoint of a 50% reduction in proteinuria without a decline in estimated glomerular filtration rate (eGFR) (45).

It has been argued that this lack of response might have been due to the already established advanced-stage FSGS lesions at the time of treatment and that further studies are required in the early phase of the disease.

Taken together, the identification of CLCF-1 as a potential circulating permeability factor is still a disputed matter. Nevertheless, it is crucial to validate its pathophysiological role in meticulously characterized patient cohorts and through independent investigations conducted by various research groups.

## 8 B cells and autoantibodies

As previously discussed, extensive research has been dedicated to investigating the pathogenesis of INS, with a focus on identifying potential permeability factors produced by T cells, following the publication of Shalhoub’s influential paper (112). However, in the past decades, compelling evidence has emerged suggesting the implication of a dysregulated B cell function in the genesis and maintenance of the disease.

In a brilliant 2017 paper, Dossier et al. provided a point-to-point rebuttal of the main pieces of evidence that supported Shalhoub’s hypothesis, ultimately proposing INS as a primary B cell disease. First of all, the authors highlighted that measles infection can lead to remission of INS as a result of impaired synthesis of immunoglobulins. Additionally, the therapeutic efficacy of steroids and cyclophosphamide can be attributed to their ability to induce apoptosis in mature B-cells. Furthermore, Reed Sternberg cells observed in Hodgkin’s disease display VDJ rearrangements in the immunoglobulin locus and express numerous B-cell surface markers. Lastly, they underscored that the antibody response to polysaccharide pneumococcal antigens relies on the B cell receptor (BCR) rather than being T cell-dependent (120).

Moreover, as opposed to Shalhoub’s and many others’ perceptions of a lack of humoral immunity involvement, recent studies have identified several autoantibodies as potential mediators of podocyte injury in INS. For instance, anti-ubiquitin C-terminal hydrolase L1 (UCHL1) IgG, an autoantibody targeting podocytes, has been recognized in a subgroup of INS patients (162). Watts et al. recently discovered circulating anti-nephrin antibodies in 29% of patients with MCD, which showed a significant reduction following treatment response. The presence of these autoantibodies correlated with positive podocyte-associated IgG staining in renal biopsies (163). Furthermore, the development of a panel consisting of seven circulating antibodies has demonstrated an impressive 92% accuracy in predicting post-transplant FSGS recurrence among a cohort of 64 patients (164). In a similar experiment, Ye et al. demonstrated that 66% of children with INS exhibited circulating podocyte autoantibodies, which correlates with higher 24-h proteinuria levels (165).

Nowadays, the main proof supporting the involvement of B cells in the pathogenesis of INS is provided by the demonstrated effectiveness of the treatment with rituximab, an anti-CD20 monoclonal antibody that selectively depletes B cells, in patients with steroid-dependent or frequently relapsing INS (166).

Providing additional support for the involvement of B cell activation in INS, it is widely recognized that mesangial deposits of low or moderate levels of IgM are frequently observed in patients with FSGS (167, 168). Furthermore, elevated CD19^+^ and CD20^+^ B cell counts have been observed during relapses of NS in patients with steroid-sensitive forms of the disease, followed by subsequent reductions upon achieving remission (169). However, conflicting data exist regarding this matter (170).

### 8.1 B cell immunophenotypes

Recent studies focused on identifying specific immunophenotypes of B cells involved in the pathogenesis of INS.

Studies conducted on pediatric patients have revealed significant differences in the composition of B cell subsets between SSNS and SRNS patients, as well as healthy volunteers, during the initial onset and relapse stages. Specifically, during relapses of SSNS, there was a notable increase in memory B-cells (CD27^+^), primarily driven by elevated proportions of IgM-memory B-cells (CD27^+^IgD^+^IgM^+^) and switched-memory B-cells (CD27^+^IgD^-^IgM^-^). In contrast, transitional B-cells (CD19^+^CD24^hi^CD38^hi^) were significantly elevated in SSNS compared to SRNS patients. Notably, the proportion of transitional B-cells proved effective as a biomarker for predicting the response to prednisone therapy, with a cutoff value of 2.05 (% of total lymphocytes) enabling the differentiation of SSNS from SRNS with a sensitivity of 79.1% and a specificity of 90.9% (171). The same group demonstrated that a lower transitional B-cell proportion and a higher memory B-cell proportion were associated with an increased risk of disease relapse during a one-year follow-up in SSNS patients who had a stable response to steroid treatment. They, therefore, proposed a low transitional B-cell to memory B-cell ratio as an independent risk factor for recurrence-free survival in SSNS patients (172).

Similarly, Colucci et al. documented significantly higher values of total memory B-cells and switched memory B-cells (but not IgM-memory B-cells) during relapse compared to control onset and remission values, in pediatric patients with SSNS (173).

Moreover, switched memory B-cell recovery after rituximab treatment was significantly delayed in non-relapsing pediatric INS patients compared to the relapsing group, proving to be a valuable predictor of treatment response (174).

Additional investigations conducted by Fribourg et al., utilizing Time-of-flight mass cytometry analysis, confirmed that switched memory B-cells constitute the primary lymphocyte subset associated with disease relapse in children suffering from steroid-dependent INS (175).

Limited evidence is available about B cell profile alterations in adult patients with NS. Among 22 adult patients with active MCD and NS, plasmablasts (CD24^-^CD38^high^CD27^high^) were the only B cell subset found at a significantly higher level compared to patients in remission, patients with idiopathic membranous nephropathy, and healthy controls. Plasmablasts levels positively correlated with lower albumin and higher proteinuria levels. Moreover, increased levels of IL-21, IL-6, and B-cell activating factor (BAFF) in the serum of relapsing patients were significantly correlated with the percentage of plasmablasts (176). In a recent study conducted by Casiraghi et al., an elevation in the memory B-cell population was observed in adult patients with steroid-dependent/frequently relapsing NS (SDNS/FRNS) and SRNS compared to healthy volunteers. SDNS/FRNS patients predominantly exhibited a CD38-negative phenotype, while SRNS patients were characterized by a switched phenotype (140).

In the past decade, an intriguing hypothesis linking chronic viral infections to the immunopathogenesis of INS has gained attention. One prominent aspect of this hypothesis focuses on the potential role of EBV latency in memory B-cells. Specifically, it is hypothesized that anti-Epstein-Barr nuclear antigen 1 (EBNA1) antibodies may exhibit cross-reactivity with major podocyte proteins following their internalization through the neonatal Fc receptor. Consequently, this process is suggested to contribute to the development of the histological and clinical characteristics associated with INS (120).

Interestingly, Colucci et al. observed the production of hypo-sialylated IgM antibodies that bind to T cell surfaces in pediatric patients with INS and poor response to therapies, suggesting a potential mechanism for steroid dependence in the disease. This finding implies the existence of a pathogenic connection between B cells and T cells in MCD and FSGS (177). Finally, IgM has been shown to activate the classical pathway of complement in the glomeruli of INS patients, contributing to the damage (178).

These findings shed light on the intricate involvement of dysregulated B cell function and the presence of autoantibodies in the pathogenesis of INS. However, further investigations are required to elucidate the specific mechanisms by which B cells contribute to the disease and their interaction with T cells and complement activity.

## 9 B-cell activating factor in circulating factor disease

The B-Cell activating factor (BAFF), also known as tumor necrosis factor ligand superfamily member 13B (TNFSF13B) or B Lymphocyte Stimulator (BLyS), is a homeostatic cytokine belonging to the TNF family. BAFF is expressed by various cell types in the immune system, such as monocytes, activated neutrophils, T cells, and dendritic cells. Initially expressed as a membrane protein, BAFF can be released into circulation after processing and cleavage. Its expression and secretion are upregulated during inflammation in response to different stimuli, including IL-2, TNF-α, and interferon- γ (IFN-γ) (179).

BAFF interacts with three distinct receptors, mainly expressed by B lymphocytes: BLyS receptor 3 (BR3) or BAFF-R, transmembrane activator–1 and calcium modulator and cyclophilin ligand–interactor (TACI), and B-cell maturation antigen (BCMA). These interactions play a critical role in the selection, survival, and maturation of B cells. Specifically, the interaction between BLyS and its receptor, BR3, is essential for the viability of newly formed and mature primary B cells. This interaction provides vital and nonredundant survival signals necessary for their continued existence (180). Interestingly, BAFF actively participates in supporting B-cell survival during the early stages of B-cell maturation and the differentiation of B cells into antibody-producing plasma cells, whereas the levels and function of memory B cells are independent of BAFF (181).

BAFF activity specifically plays a crucial role during the transitional stages of B cell differentiation. This differentiative stage is the major peripheral checkpoint for the elimination of potentially autoreactive B cells before their maturation. An excessive amount of BAFF can disrupt this process, allowing less avid self-reactive clones to evade anergy and be rescued, thereby leading to a breakdown in B-cell tolerance and the development of autoimmunity (179, 182).

Elevated levels of BAFF have been observed in the serum of patients with various autoimmune diseases, such as rheumatoid arthritis (183), Sjögren’s syndrome (184), or systemic lupus erythematosus (SLE) and lupus nephritis (179). A variant in the TNFSF13B gene, which encodes for BAFF, has been associated with increased serum levels of this cytokine and has been linked to multiple sclerosis and SLE (185).

In the kidney, studies have reported higher levels of BAFF in direct relation to the activity of IgA nephropathy (186), while limited information is currently available regarding the association between BAFF and INS, particularly in FSGS patients.

BAFF expression on kidney biopsy was associated with a more rapid decline in GFR and overall lower GFR values in a cohort of 33 pediatric patients with MCD or FSGS and NS. Podocytes and interstitial inflammatory infiltrates were found to express BAFF in 18.2% and 36.4% of the biopsy samples, respectively (187). Belimumab, a monoclonal antibody targeting BAFF, has shown efficacy in the treatment of various autoimmune conditions and it is approved for the treatment of refractory SLE, including lupus nephritis (188). Belimumab has also demonstrated promising results in reducing anti-HLA antibody titers in kidney transplant humoral rejection (189) and in reducing anti-phospholipase A2 receptor autoantibodies (PLA2R-Ab) and proteinuria (both important hallmarks of the disease) in primary adult membranous nephropathy (190).

In a recent phase two trial, treatment with belimumab in ten frequently-relapsing NS pediatric patients was evaluated. Although the treatment was well-tolerated, the study was terminated after the interim evaluation as researchers did not find clear improvements in the mean number of relapses and mean prednisone dose, in a six-month period of observation. It is important to note that the small number of patients and the short follow-up period limited the ability to draw definitive conclusions regarding efficacy. Nonetheless, it is important to consider the burden of monthly in-hospital intravenous infusions in this therapeutic scheme, as it can outweigh the potential benefits. Notably, belimumab therapy was associated with a decrease in transitional and mature-naive B-cells, while memory B-cells were not significantly affected, supporting their potential role in the pathogenesis of the disease (191).

To sum up, the role of BAFF in the pathogenesis of INS and permeability factor-related FSGS is still not fully understood. As it holds promising potential, further studies are needed to unravel its precise contribution to the development of the disease and its recurrence after kidney transplantation.

## 10 Role of the complement system in circulating factor disease

Segmental deposition of C3 in the sclerotic portion of the glomerular tuft, as well as occasionally in the adjacent mesangium and unaffected glomeruli, represents well-established features present in a significant percentage of FSGS patients (168, 192) The significance of these deposits, frequently associated with IgM deposits, is uncertain.

Studies in rodent models of FSGS, such as adriamycin nephropathy, observed increased deposition of the terminal complement complex C5b-9 within the kidney, particularly on the apical surface of proximal tubular cells and in the peritubular region. Adriamycin-treated C6-deficient rats, which cannot form C5b-9, demonstrated reduced interstitial extracellular matrix deposition, less tubule-interstitial injury, minor peritubular myofibroblast accumulation, and reduced interstitial monocyte infiltration compared to C6-sufficient rats (193), supporting the role of the terminal complement activation in disease progression.

Furthermore, in the same model of FSGS, promising results have been observed by inhibiting decay-accelerating factor (DAF) cleavage on podocytes, a key complement regulator, through both genetic and pharmacological approaches. These interventions have shown potential in preventing the onset and progression of FSGS (194).

In a different mouse model of NS, induced by the protein-overload, abnormal fixation of ultrafiltered C3 was detected in tubuli and podocytes showing signs of injury and dedifferentiation during the early stage of the disease. Moreover, C3-deficient mice with protein overload were protected against podocyte structural damage and sclerosis, indicating that complement activation might increase susceptibility to injury (195).

Interestingly, mutations in factors H and C3 have been described in literature cases of biopsy-proven FSGS (196). Moreover, in a cohort of 19 patients with FSGS, plasma and urine levels of complement activation components Ba, C4a, and sC5b-9 were significantly higher than in control patients with CKD, ANCA vasculitis, lupus nephritis, or in healthy controls. These findings suggest a potential pathogenetic role of complement activation in the development of FSGS. Notably, plasma Ba levels exhibited an inverse correlation with the eGFR at the time of diagnosis and the end of the study. Additionally, plasma and urine Ba levels at the end of the study showed a positive correlation with the level of proteinuria, which was the primary outcome of the study (197). In a similar study by Huang et al., the authors demonstrated elevated levels of C3a, C5a, and C5b-9 in both the plasma and urine of FSGS patients compared to normal controls. The levels of plasma and urinary C5b-9 were positively correlated with urinary protein, renal dysfunction, and interstitial fibrosis. Furthermore, plasma C5a levels showed a positive correlation with the proportion of segmental sclerotic glomeruli (198). Recently, among a cohort of 112 autoimmune glomerulonephritis patients, urines from patients with either MCD or FSGS showed significantly increased levels of C5a, which directly correlated with the severity of proteinuria (199).

## 11 Crosstalk between complement and adaptive immunity

The association between complement system and adaptive immunity in the pathogenesis of FSGS has been extensively demonstrated in numerous studies.

For instance, when BALB/c mice deficient in DAF were injected with sheep anti-mouse podocyte antibodies, they exhibited histological and ultrastructural characteristics of FSGS, marked albuminuria, periglomerular monocytic and T cell infiltration, and heightened T cell reactivity to sheep IgG. Notably, depleting CD4^+^ T cells from DAF-deficient mice led to a substantial reduction in all of these observed features, suggesting that signals derived from the complement system may influence T cell response and contribute to the development of FSGS (200).

Furthermore, recent research has shown an emerging interest in understanding the interplay between the complement system and B cell immunity in primary FSGS. This relationship has been initially established in the rodent adriamycin nephropathy model of FSGS. In this model, complement activation within the glomerulus was mediated by IgM and contributed to disease progression (201). Confirming those pre-clinical studies, Trachtman et al. demonstrated elevated levels of C4a and C5b-9, along with increased levels of self-reactive IgM, in the plasma of patients with INS compared to healthy control subjects. Similarly, they observed co-localization of IgM with activated complement fragments C4d, iC3b/C3d, and C9neo within certain glomeruli of individuals with FSGS. Based on these results, they suggested that IgM activates the complement system in the glomeruli of some patients with INS, contributing to injury (178).

Furthermore, anaphylatoxins C3a and C5a may be important mediators of the complement system-B cell immunity crosstalk. Anaphylatoxins are potent chemotactic factors generated in the complement activation cascade through the enzymatic cleavage of C3 and C5 by convertase enzymes (202). They serve as a powerful chemoattractant, guiding the directed migration of various immune cells. Among these cells, neutrophils and macrophages are particularly responsive to anaphylatoxins, which trigger the synthesis and release of inflammatory mediators such as TNF, IL-1, IL-6, CC chemokines, and CXC chemokines, perpetuating the inflammatory cascade (203).

Recent literature has highlighted an interesting novel role of BAFF in the interplay between the complement system and B cells in the adaptive immune system activity. This crosstalk is mediated by the activity of the neutrophils, which express complement receptors and can release BAFF upon activation (204). A noteworthy 2023 study by Cumpelik et al. demonstrated the existence of a T cell-independent B cell response that involves alternative pathway complement activation and the presence of neutrophil-expressed C3a and C5a receptors (C3aR1/C5aR1). These receptors promote BAFF-dependent expansion of B1-cells and T cell-independent antibody production. Notably, the conditional absence of C3aR1/C5aR1 on neutrophils resulted in lowered serum BAFF levels. These findings indicate that sequential complement activation on neutrophils critically supports humoral T cell-independent responses by upregulating neutrophil production of BAFF (205). Given the solid evidence supporting the role of both the complement and B cells, pathogenetic mechanisms of post-transplant FSGS recurrence may involve primary complement system activation, which stimulates excessive BAFF secretion by neutrophils, resulting in a B cell-mediated damage to the glomerular barrier.

Additionally, Paiano et al. (206) uncovered further insights into the involvement of complement in B2-cell responses. Their experiments revealed that signaling through C3aR1/C5aR1 is necessary for B2-cell responsiveness to BAFF and for multiple stages of B2-cell activation essential for class switch recombination and affinity maturation. These findings highlight the complexity of the role of anaphylatoxins in both B1-cell and B2-cell responses, underscoring the importance of complement-mediated signaling in the control of adaptive immune processes.

**Figure 3:**
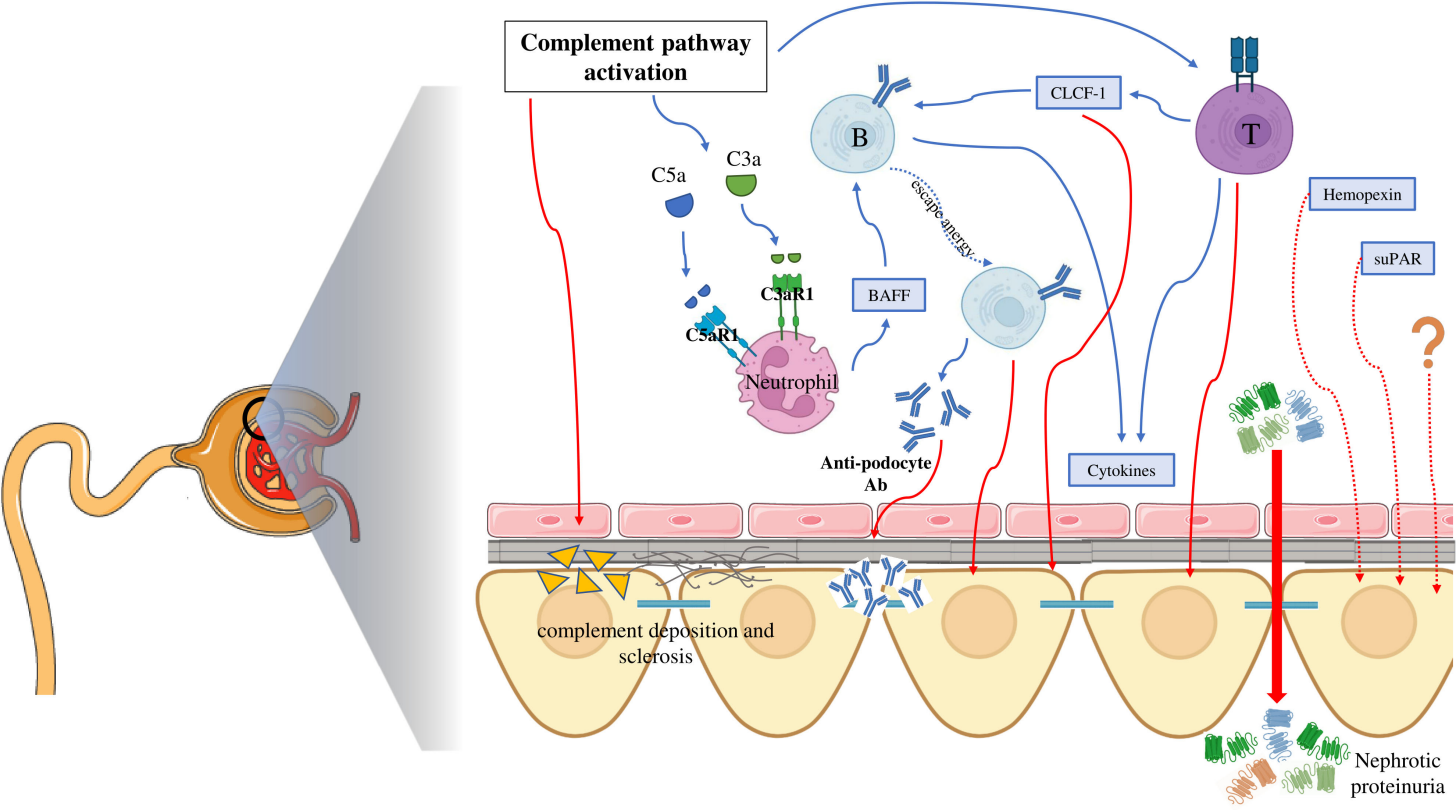
Summary of the interplay between molecular mechanisms underlying the pathogenesis of focal segmental glomerulosclerosis. C5aR1, complement 5a receptor 1; C3aR1, complement 3a receptor 1; BAFF, B cell activating factor; CLCF-1, Cardiotrophin-Like Cytokine Factor-1; Ab, antibodies; suPAR, soluble urokinase plasminogen activator receptor.

Altogether, the data described in this section support the hypothesis that the complement system plays a significant role in the pathogenesis of podocyte injury and loss in FSGS. This involvement may occur through its direct damaging effects on podocytes but there is a raising interest concerning its mechanisms of interaction with the adaptive immune system (**Figure 3**). These interactions may help elucidate the complex involvement of all these components in the development and post-transplant recurrence of FSGS.

## 12 Other (potential) circulating factors

Several other molecules have been implicated in the pathogenesis of FSGS and INS. Among these molecules, the roles of hemopexin and soluble urokinase plasminogen activator receptor (suPAR) have been extensively debated in the literature, but not conclusively confirmed. In more recent literature, other potential circulating factors have been described, including cMaf-inducing protein (cMip), CD40L, and Angiopoietin-like-4 (Angptl4).

### 12.1 Hemopexin

Hemopexin, a β1 plasma glycoprotein with a high affinity for heme, is mainly expressed in the liver and belongs to the family of acute-phase proteins, whose synthesis is induced following an inflammatory event (207).

Experimental studies in rats have shown that the injection of hemopexin triggers proteinuria and promotes the effacement of glomerular foot processes (208). This effect is believed to be mediated by hemopexin’s ability to induce nephrin-dependent reorganization of the actin cytoskeleton, leading to alterations in glomerular permeability (209). Further *in-vitro* experiments showed that glomerular mesangial cells derived from healthy individuals can produce hemopexin after prior incubation with TNF-α (210). After an almost two decades long gap in research on this topic, a study by Pukajło-Marczyk et al. in 2021 reported significantly higher serum and urine levels of hemopexin in children with INS compared to healthy controls (211). Furthermore, Agrawal et al. developed a biomarker panel able to predict SRNS at the time of disease presentation, through proteomic analysis of pediatric NS patient plasma samples. This panel included hemopexin along with 13 other biomarkers (212). Additionally, a 2022 study by Chebotareva et al. analyzed the urinary proteome profile of patients with INS using mass spectrometry. They found elevated expression of hemopexin in patients with severe progressive FSGS, compared to those with mild FSGS forms or MCD (213).

Further research is therefore needed to provide more definitive evidence regarding the role of hemopexin as a mediator or prognostic marker in FSGS.

### 12.2 Soluble urokinase plasminogen activator receptor

The role of soluble urokinase plasminogen activator receptor (suPAR) in human FSGS has been the center of a complex debate between scientists for many years and continues to be so.

suPAR refers to the soluble form of the urokinase-type plasminogen activator receptor (uPAR), which is a membrane protein linked to glycosyl-phosphatidylinositol found on various immunologically active cells. uPAR plays a role in the regulation of cell adhesion and migration by binding urokinase-type plasminogen activator, vitronectin, and integrins. suPAR originates from cleavage and release of the membrane-bound uPAR, and its levels in the serum are directly proportional to the activation of the immune system. Elevated levels of suPAR have been observed in various clinical conditions, including systemic inflammation and malignant diseases (214).

In the context of glomerular diseases, a seminal study by Wei et al., in 2008, demonstrated in mice lacking uPAR that treatment with suPAR or its overexpression led to increased signaling of αvβ3 integrin in podocytes, resulting in foot-process effacement and proteinuria (215). The same group subsequently conducted a significant study revealing elevated serum levels of suPAR in two-thirds of individuals with primary FSGS, but not in those with other glomerular diseases. They further established that higher pre-transplant suPAR concentrations were associated with an increased risk of FSGS recurrence post-transplantation. During their study, the authors established a cutoff value of 3000 pg/mL, indicating a high probability of diagnosing FSGS (216).

The discovery of the possible involvement of suPAR in the pathogenesis of FSGS sparked numerous preclinical and clinical studies, yielding conflicting results and raising doubts about the specificity of the correlation between suPAR and the disease (217).

Conflicting research findings have revealed that suPAR levels do not possess the capability to differentiate between patients diagnosed with FSGS and those affected by other glomerular diseases, including MCD, membranous nephropathy, IgA nephropathy, lupus nephritis, or non-chronic kidney disease (218–221). This suggests that suPAR might serve merely as a nonspecific marker of glomerular distress. Additionally, some studies have indicated that suPAR levels and eGFR held an inverse correlation in different patient cohorts, including those with chronic glomerular diseases, idiopathic nephrotic syndrome (INS), and particularly FSGS patients. This correlation is thought to be associated with impaired renal excretion rather than suPAR’s role as a disease biomarker (219, 222). Multiple other studies showed that serum suPAR was not useful either as a diagnostic or treatment response marker in MCD patients (223, 224).

It should be highlighted, however, that some authors supported the potential value of serum suPAR as a predictor for post-transplant FSGS recurrence (225, 226). Even in this context, the evidence is low and further research would be needed to define a correlation between serum suPAR and post-transplant recurrence of FSGS.

Therefore, the role of suPAR or any of its specific glycosylated cleavage products as a specific marker of FSGS or potential mediator of the disease has yet to be established.

### 12.3 Others

Various other molecules that could act as circulating factors and contribute to the pathogenesis of circulating factor disease have been proposed in the last decade. However, their roles in the disease are yet to be established.

In 2010, Zhang et al. reported elevated expression of Cmaf-inducing protein (cMip) in the podocytes of patients with INS. They also observed that transgenic mice overproducing cMip in podocytes developed proteinuria without visible pathologic alterations. The researchers demonstrated that cMip interacts with the Src kinase Fyn, leading to the inhibition of nephrin and N-WASP phosphorylation, resulting in cytoskeleton disorganization (227). Recent findings suggest that cMip may induce proteinuria in podocytopathies by downregulating Wilms tumor 1 (WT1) at the mRNA and protein levels, as WT1 is essential for podocyte integrity (228).

Angiopoietin-like-4 (Angptl4), a glycoprotein highly expressed in the liver and adipose tissue, was first shown by Clement et al. in 2011 to be glucocorticoid-sensitive and highly upregulated in the serum and podocytes of experimental models of MCD and human disease. In a transgenic mouse model, Angptl4 secreted specifically by podocytes was responsible for inducing nephrotic-range proteinuria, loss of glomerular basement membrane charge, and foot process effacement – all hallmarks of MCD (229). Further studies from the same group supported a potential role for Angptl4 in proteinuria development in MCD patients (230, 231). However, a larger study by Cara-Fuentes et al. in 2017, which included 60 MCD and 52 FSGS patients, did not confirm these findings and found lower serum Angptl4 levels in patients with MCD, FSGS, and membranous nephropathy during relapse compared to controls. The study did find increased urinary Angptl4 levels in MCD patients, but they were similar to those observed in patients with massive proteinuria due to other glomerular diseases (232).

CD40, along with its ligand, CD40L, belongs to the tumor necrosis factor receptor (TNFR) superfamily and plays a role in the maturation and activation processes of multiple immune cell lines, exerting pro-inflammatory effects (233). At the kidney level, glomerular epithelial cells constitutively express CD40. Blocking the CD40-CD40L interaction has shown protective effects in animal models of FSGS (78, 234). A soluble form of CD40L (sCD40L), resulting from proteolytic cleavage of CD40L, has been detected in circulation and associated with a potential pathogenetic role in primary FSGS. Doublier et al. demonstrated that sCD40L could bind to CD40 on the membrane of glomerular epithelial cells, leading to an alteration of nephrin and podocin distribution both *in vivo* and *in vitro*. Inhibition of CD40-CD40L interaction *in vitro* prevented these effects. Furthermore, the study found significantly elevated levels of sCD40L in the sera of adult patients with biopsy-proven FSGS compared to healthy subjects (235). It is worth mentioning that the previously mentioned study by Delville et al. also detected anti-CD40 antibodies in the serum of patients with recurrent FSGS, which alone predicted post-transplant recurrence with 78% accuracy (164).

In conclusion, although these findings hold promise, further research is needed to definitively confirm or exclude the role of these molecules in the pathogenesis of primary FSGS.

## 13 Animal models of FSGS

Several animal models resembling FSGS have been developed in the last decades. All these models induced podocyte injury that was caused by toxic agents or nephron reduction, by a combination of genetic mutation and toxins or by specific podocyte gene disruption.

Adriamycin (236–241) and puromycin aminonucleoside (242–248) are the podocyte toxin drugs most used to induce FSGS. When administered to susceptible rodent strains, they induced progressive glomerular disease, proteinuria, and development of histologic FSGS within few months.

Models of inducible FSGS have been also created to allow controlling the onset and severity of podocyte-specific injury. The Thy-1.1 model was generated by inducing podocyte expression of a hybrid human-mouse Thy-1.1 antigen. Ectopic expression of Thy-1.1 induced per se spontaneous development of proteinuria and histological lesions of FSGS in some glomeruli within 6 months of age (249). Injection of anti Thy-1.1 antigen antibody induced acute proteinuria and accelerated the development of FSGS (250, 251). Similarly, transgenic expression of the diphtheria toxin receptor (252) or of human CD25 (253) in podocytes allowed inducing defined levels of podocyte depletion by titrating the administration of diphtheria toxin in rats (252) or the LMB2 immunotoxin with specific binding to human CD25 in NEP25 mice (253, 254), respectively.

These models have been fundamental to demonstrate that podocyte injury caused FSGS in a dose-dependent manner, however they do not address the cause of primary FSGS.

To dissect the role of specific podocyte proteins whose mutations have been identify as a monogenetic cause of nephrotic syndrome, several knock-out and knock-in murine transgenic models have been created. So far murine models transgenic for slit diaphragm and cytoskeleton of the foot process protein genes, such as NPHS1 (255, 256), NPHS2 (257, 258), αACT4 (259, 260), CD2AP (261), TRPC6 (262), PODXL (263) are available and provided invaluable insights into the function of the single protein and the role of its abnormality in the pathogenesis of proteinuria and FSGS development in the genetic forms of the disease.

All the above models utilizing either chemical or genetic podocyte depletion strategies led to important gain of knowledge of the complex pathophysiology of FSGS but do neither involve a circulating factor nor replicate the underlying immunologic abnormalities. Therefore, these models are not suitable for studying the pathologic process of human primary FSGS.

The model of spontaneous FSGS in Buffalo/Mna rat might be considered a primary FSGS-like disease induced by a circulating factor based on proteinuria recurrence on the transplanted healthy kidney in these rats (264, 265). The development of nephrotic syndrome in Buffalo/MWF is preceded by kidney infiltration of activated macrophages and T cells and by Th2 polarization (266), suggesting that primary FSGS in this model could be an immune-mediated disorder. Nevertheless, the slower pace of kidney deterioration has hampered the use of this model in FSGS research.

In an effort to create an *in vivo* model to detect the presence of circulating permeability factor in FSGS patient blood samples, den Braanker at al (267)., backcrossed the Thy-1.1 transgene across five mouse strains and identified Balb/cThy-1.1 and C57BL/6 Thy-1.1 mice as FSGS-prone models suitable for testing circulating permeability factor. Injection of plasmapheresis effluent from FSGS patients, at a dose that did not induce protein overload, accelerated albuminuria development in these FSGS-prone mice. However, the injection of serum and plasma samples from the same patients failed to induce proteinuria, limiting the applicability of this model for diagnostic purpose as well as its value as research model.

Recent studies highlight the potential of the recently developed humanized mouse models as a tool to investigate the immunologic abnormalities in FSGS. In a study of Sellier-Leclerc et al. (268), the injection of immature CD34+ cells isolated from blood of patients with MCD and FSGS - but not the injection of CD34- cells or cells isolated form healthy volunteers - into humanized NOD/SCID mice induced proteinuria and typical FSGS renal lesion at electron microscopy. The engraftment of CD34+ cells from NS patients in humanized mice paralleled the increase in albuminuria, whereas injection of mature T cells from the same subjects did not induce any changes in urinary albumin-to-creatinine ratio. The authors suggested that the cells responsible of glomerular injury were immature cells undergoing differentiation (268). A more recent study implicated BM-derived immature myeloid cells as cells responsible for the development of proteinuric kidney diseases, including FSGS (269). Through bone marrow transplantation studies in uPAR deficient and sufficient mice, the authors identify Gr-1low immature myeloid cells as the source of elevated levels of suPAR in proteinuric animals. These cells were able to induce proteinuria when injected into healthy mice. Injection of whole PBMC – but not CD34-depleted PBMC – derived from patients with recurrent FSGS into humanized NSG mice resulted in Gr-1low myeloid cell expansion in the BM, higher suPAR levels and development of proteinuria, suggesting that FSGS might be considered a hematopoietic stem cell disorder.

These humanized mice models are promising useful tools to study the involvement of stem and immune cells in FSGS pathogenesis and will be instrumental for future studies aimed at identifying the cellular source of the circulating permeability factor.

Overall, the lack of animal models resembling primary FSGS has represented a main obstacle in the research and is still hampering our ability to get insight into the disease pathogenesis and treatment. However, important advances through new technologies, such as humanized mice (268, 269), zebrafish model system (270), *in vitro* kidney organoid and 3D coculture (271) are expected in the coming decade.

## 14 Discussion and future directions

In conclusion, the pathogenesis of post-transplant disease recurrence in patients with primary FSGS remains a complex and incompletely understood process. Various authors agree that circulating factor disease may encompass a wide spectrum of disorders, each characterized by distinct pathologic mechanisms. In each subtype, multiple specific mechanisms may contribute to the podocyte dysfunction and abnormal glomerular permeability observed, as well as their recurrence following kidney transplantation (104).

A critical limitation of current studies on circulating factor-related FSGS is the inclusion of patients without prior transplantation, whereas it is only the forms of FSGS that rapidly recur after kidney transplantation that can be confidently attributed to a circulating permeability factor. To validate the pathophysiological role of specific molecules in FSGS, it is imperative to establish well-characterized patient cohorts, particularly focusing on smaller cohorts consisting exclusively of post-transplant recurrent FSGS patients. Thus, to enhance the significance and scientific validity of studies on the pathogenesis of primary FSGS, the establishment of international biobanks should be encouraged, aimed at increasing the sample size of studies that adopt this more precise patient inclusion criterion.

The existing hypotheses only offer partial explanations for the pathogenesis of primary FSGS. It is proposed that the pathogenesis may involve a multi-hit process, wherein the various molecules proposed as potential unique circulating factors interact with one another and with other unidentified factors, inducing the first manifestation of proteinuria and triggering a self-perpetuating mechanism (110). Furthermore, it is possible that a missing circulating molecule, rather than an excess of one, is involved in the pathogenesis of FSGS.

Each candidate molecule and pathogenetic mechanism may not exclude the complementary pathogenic role of others, including those that are yet unknown. Therefore, further research should adopt a modified approach by focusing on better-characterized smaller patient cohorts, as mentioned above, and incorporating high-throughput hypothesis-generating techniques such as -omics technologies. This approach holds great promise for unraveling the intricate complexity of immune response and imbalances underlying circulating factor-related diseases, aiming to uncover their intersections and develop a comprehensive analysis of primary FSGS alterations, leading to a global understanding of the disease.

Furthermore, a notable challenge that demands attention is the absence of an *in vivo* preclinical model that demonstrates a pathological response upon exposure to serum or plasma from primary FSGS patients. The development of such a model would be of paramount importance, as it would be crucial for validating the pathogenetic role of candidate circulating factors and would pave the way for studies aiming to identify the active fraction of plasma from primary FSGS patients, similar to Savin’s *in-vitro* research (155).

Adopting this comprehensive top-down and bottom-up approach, encompassing both hypothesis testing and hypothesis generation, would be crucial in definitively identifying the underlying cause of the disease.

## 15 Conclusions

In summary, the pathogenesis of post-transplant disease recurrence in primary FSGS requires further investigation and understanding. Establishing well-characterized patient cohorts, specifically focusing only on post-transplant recurrent FSGS patients, will contribute to validating the role of specific molecules in the disease. The convergence of hypotheses and the implementation of high-throughput techniques, including single-cell-based approaches, offer promising avenues for comprehending the complex immune response and imbalances underlying circulating factor-related FSGS. Additionally, the development of an *in vivo* preclinical model would be crucial in assessing the pathogenicity of serum or plasma from FSGS patients, ultimately advancing our knowledge of this challenging condition.

## Author contributions

GS and FC wrote the manuscript. FC and GR played a key role in editing and revising the manuscript for clarity and coherence. All authors contributed to the article and approved the submitted version.
